# Biomechanical comparison of vertical suture techniques for repairing radial meniscus tear

**DOI:** 10.1186/s40634-020-00296-w

**Published:** 2020-10-06

**Authors:** Guanqi Hang, Andy Khye Soon Yew, Siaw Meng Chou, Yoke Rung Wong, Shian Chao Tay, Denny Tijauw Tjoen Lie

**Affiliations:** 1 Department of Orthopaedic Surgery, Singapore General Hospital, 20 College Road, Singapore, 169865 Singapore; 2 School of Mechanical & Aerospace Engineering, College of Engineering, Nanyang Technological University, 50 Nanyang Ave, Singapore, 639798 Singapore; 3 Department of Hand Surgery, Singapore General Hospital, 20 College Road, Singapore, 169865 Singapore

**Keywords:** Radial tear, Meniscus repair, Medial meniscus, Mattress suture, Arthroscopy

## Abstract

**Purpose:**

The aim of this study was to (1) develop suture techniques in repairing radial meniscal tear; (2) to compare the biomechanical properties of the proposed repair techniques with the conventional double horizontal technique.

**Methods:**

Thirty-six fresh-frozen porcine medial menisci were randomly assigned into four groups and a complete tear was made at the midline of each meniscus. The menisci were subsequently repaired using four different repair techniques: double vertical (DV), double vertical cross (DVX), hybrid composing one vertical and one horizontal stitch, and conventional double horizontal (DH) suture technique with suturing parallel to the tibia plateau. The conventional double horizontal group was the control. The repaired menisci were subjected to cyclic loading followed by the load to failure testing. Gap formation and strength were measured, stiffness was calculated, and mode of failure was recorded.

**Results:**

Group differences in gap formation were not statistically significant at 100 cycles (*p* = .42), 300 cycles (*p* = .68), and 500 cycles (*p* = .70). A trend was found toward higher load to failure in DVX (276.8 N, *p* < .001), DV (241.5 N, *p* < .001), and Hybrid (237.6 N, *p* < .001) compared with DH (148.5 N). Stiffness was also higher in DVX (60.7 N/mm, *p* < .001), DV (55.3 N/mm, *p* < .01), and Hybrid (52.1 N/mm, *p* < .01), than DH group (30.5 N/mm). Tissue failure was the only failure mode observed in all specimens.

**Conclusion:**

Our two proposed vertical suture techniques, as well as the double vertical technique, had superior biomechanical properties than the conventional technique as demonstrated by higher stiffness and higher strength.

## Background

The knee meniscus plays a key role in joint lubrication, shock absorption, load-bearing and load distribution, thus it often withstands different forms of forces and is susceptible to injuries [2, 3, 26, 42]. Radial meniscal tear has increasingly become common, particularly in sports traumatology [28]. In the last century, partial or total meniscectomy – removing the damaged meniscal tissue - was the gold standard to treat meniscal tear [14, 19]. However, joint deterioration and early onsite of knee osteoarthritis were reported in studies following up patients underwent meniscectomy treatment [25]. As a result, it is widely accepted today that treatment of meniscus tear should preserve the knee meniscal tissue as much as possible [18, 22, 23, 28].

The inside-out/outside-in technique passing horizontal stitches has been commonly used today to treat various types of meniscal tear, however, it is still inconclusive whether this method is the optimal approach to repair radial meniscal tear [4, 6, 20, 24, 27, 33]. In light of the limitations of the traditional inside-out double horizontal suture used for repairing radial tears [7], novel repair approaches using horizontal suture orientations were developed and tested. For example, the horizontal butterfly technique developed by Günes et al. decreases the amount of displacement but yields a similar failure load compared to the standard horizontal loop [4]. Solitro et al. reported that their reinforced repair technique has a higher failure load than the standard repair technique with two parallel sutures [27]. Meanwhile, suture techniques deploying a vertical orientation are developed and compared to techniques using horizontal stiches [10, 16]. The single loop vertical suture, a technique developed by Beamer et al. [6], was reported to be superior in strength than the single loop horizontal suture in repairing complete radial tear. However, Lemos et al. reported that the vertical suture has a significantly lower failure load than the inside-out parallel suture [16]. As no consensus on the best approach to repairing radial meniscus tear has been made, researchers and practitioners are calling for the development of novel repair techniques and studies that compare the performance between different techniques so that evidence-based decisions on the optimal approach can be made [8, 11, 13, 40].

Furthermore, the traditional all-inside device is only able to pass horizontally suture and deploy anchors at the meniscal peripheries. As a result, the suture cannot fully encircle the tear and failure at the anchors compromises the repair [6, 24, 35]. The invention of a novel suture passer, which was used in this study, solved this problem, as it enables passing suture vertically from the under-surface of the meniscus and allows easy adjustment of the alignment of sutures relative to the circumferential fibrils.

In light of the gap in research evidence and the advancement of medical device used for orthopedic surgery, we proposed two suture techniques for repairing radial meniscal tear: the double vertical cross and the hybrid suture techniques. The double vertical cross consists of two vertical stitches forming a cross. Since it is generally more challenging to stitch vertically than horizontally, we combined one vertical and one horizontal stitches in the hybrid suture technique. Compared to the abovementioned studies, our proposed two approaches deploy vertical sutures perpendicular to the tibia and suture configurations different from the other vertical suture techniques. Additionally, we included the double vertical parallel technique in our study, which enables a more systematic comparison of vertical suture orientation to the traditional horizontal suture technique.

The objective of this study was to assess the biomechanical properties, including gap formation, strength, and stiffness, of the three vertical two-stitch suture repair techniques compared with the traditional double horizontal suture technique in complete radial tear of the medial menisci. We hypothesized that all three techniques - double vertical cross, double vertical, and hybrid composing one vertical and horizontal strand - would offer better fixation and primary stability in the repair of complete radial meniscus tear. Vertically oriented stitches contribute to the stability of repaired construct more than horizontal stitches.

## Materials and methods

### Specimen preparation and suture techniques

We conducted an in vitro biomechanical study on fresh-frozen size-equivalent porcine medial menisci. A total of 36 specimens were harvested intact from adult hogs by resecting the tissue at the meniscoscapular junction and the two insertional ligaments. All the menisci were inspected and had no macroscopic signs of meniscal tear or degeneration. The resected menisci were thawed 5 h at room temperature (23–25 degree Celsius), and then wrapped with normal saline soaked gauze before testing. Radial tears were created at the meniscal mid-body equidistant from the anterior and posterior horns with a No. 11 surgical blade. The tears extended from the central margin to 1 mm from the peripheral meniscus rim.

**Fig. 1:**
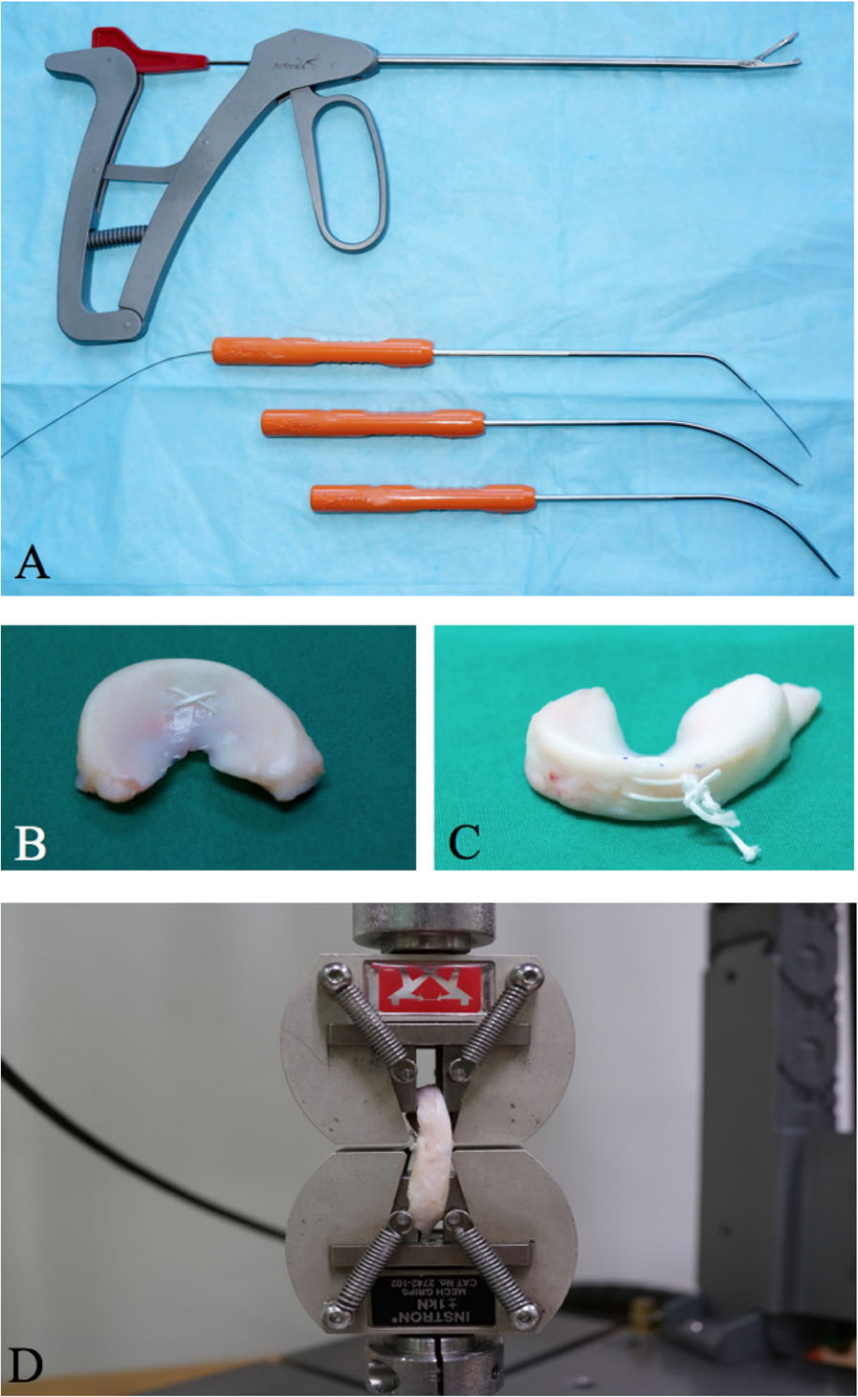
**a** Scorpion (top) and Micro SutureLasso with different curvatures (bottom) meniscus repair devices used in the study; **b** Double vertical cross repair with the Scorpion device; **c** Double horizontal repair with the Micro SutureLasso device; **c** mechanical testing set up

After preparing the menisci with tears, the 36 specimens were randomly assigned into four groups to be repaired using different techniques: double horizontal mattress (simplified as “DH”) (*n* = 9), double vertical mattress (“DV”) (*n* = 9), double vertical cross (“DVX”) (*n* = 9), and Hybrid (*n* = 9). In the DH group (Fig. 1a), the meniscal tear was repaired with two horizontal loops at a distance of 5 mm on either side of the tear. The loop on superior level was placed 4 mm from the meniscal rim and the inferior loop was stitched 6 mm from the meniscal rim. The tested menisci in DV group were repaired with two parallel sutures at 5 mm from the tear and 4 and 6 mm from the rim. In addition, the orientation of the two loops are perpendicular to the bottom surface (Fig. 1b). In the DVX group, vertically oriented sutures crossed each other and were stitched at the same fixation points as in the DV group (Fig. 1c). The Hybrid technique comprised one horizontal loop and one vertical loop (Fig. 1d). The vertical loop was stitched in vertical orientation with a distance of 5 mm from the tear and 3 mm from the rim. The horizontal loop was placed in horizontal orientation with a distance of 5 mm from the tear and 9 mm from the rim.

**Fig. 2:**
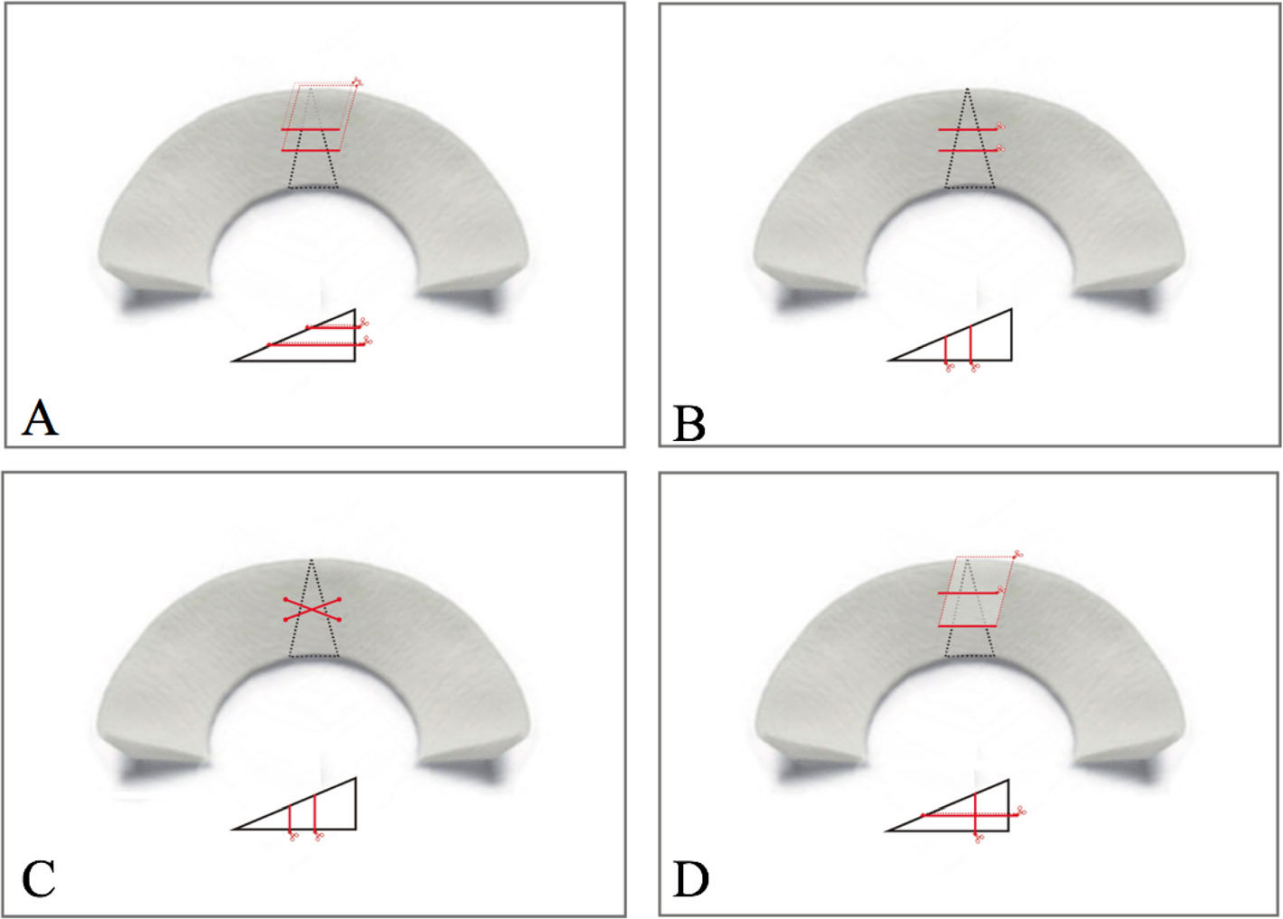
Illustration of meniscus repair techniques: **a** double horizontal technique, **b** double vertical technique, **c** double vertical cross technique, **d** hybrid technique

All the vertical stitches were made in this study using the Knee Scorpion device (Arthrex, North Naples, FL) (Fig. 1a top). All the horizontal stitches were made with the Micro SutureLasso device (Arthrex, North Naples, FL) (Fig. 2a bottom). Same suture material was used (2–0 Ultrabraid, Smith&Nephrew, Andover, Massachusetts). Knee Scorpion (Arthrex, North Naples, FL) is a novel all-inside device using an articulated jaw to hold the meniscus and pass the suture vertically.

All specimens were tied by hands in an open fashion. Four square knots were tied with four throws. Once tied, the radial tear was completed by extending the tear through the meniscal rim. Before cyclic testing, each repaired specimen was inspected for any suture or tissue damage during the repair process. Specimens with damaged suture or unsecured knots were discarded.

### Cyclic load testing

The repaired menisci’s two ends were stitched with baseball sutures using 5–0 Ethibond (Ethicon Inc., Johnson&Johnson) and then securely fastened to the universal tissue clamps with texture surface to prevent tissue slippage. The repaired menisci were aligned perpendicular to the tear, subsequently mounted to a mechanical testing system (Instron E1000, Instron, Norwood, Massachusetts) (Fig. 1d). During the pilot testing, specimens with the above-described roughening technique were tested and confirmed with negligible slippage at the interface between tissue and clamps.

The primary outcomes of our study were the ultimate failure load, stiffness and displacement during cyclic loading. After a preload of 2 N was applied to the specimen, cyclic loading from 5 to 20 N was performed at a frequency of 1 Hz and a crosshead velocity of 12.5 mm/s [6, 24, 35, 39, 41]. The load and frequency were chosen based on previous studies and reflective of in vivo post-operational rehabilitation [5, 6, 9, 12, 28, 29, 35, 37, 45, 46]. Specimens underwent 900 submaximal loading cycles and the Instron device was programed to pause 45 s at 0, 100, 300, 500 cycles to enable data collection and normal saline was sprayed to keep the specimens moist. The gap formation (displacement) was measured by the relative position of actuators. Both displacement and its corresponding load were recorded continuously by the computer program (Wavematrix; Instron, Norwood, MA). The gap formation was measured and recorded as the average distance across the tear at 12.5 N at 0, 100, 300, and 500 cycles. Cycle 0 served as the reference point for reporting the displacement of subsequent cycles 100, 300, and 500. After completion of cyclic loading, load to failure testing was performed at a rate of 3.15 mm/s. Stiffness as the linear region of the load-displacement curve was calculated. The mode of failure was carefully inspected and recorded. Three possible failure modes were tissue failure (pulled through by suture), suture failure (breakage), and knot failure (knot slippage).

### Statistical analysis

A pilot testing of three specimens per group was performed and the effect size of 0.6 was determined from the load to failure pilot data. Power analysis using that effect size was calculated using G*Power (version 3.1.9.2) [17]. Nine specimens per group were determined to be adequate to detect a 20% change in the load to failure at 80% power. One-way analysis of variance (ANOVA) with post hoc Tukey analysis was performed to assess differences in the load to failure and stiffness across the four groups and between any two groups. Two-way ANOVA was performed at different cycles (100, 300, and 500 cycles) to test the differences between the four groups and between cycles. Data analysis was performed using R (Version 3.22) [R core Team, 2015]. All comparisons were two-tailed and a *P* value <.05 was considered statistically significant.

## Results

The comparison of the primary outcomes – the ultimate failure load, stiffness, and displacement during cyclic loading – between the four groups were summarized in Table 1.

**Table 1:** Load to failure, stiffness and displacement results for the 4 repair techniques

	Double Vertical Cross	Double Vertical	Hybrid	Double Horizontal
Load to Failure, N	276.8^1,2^± 39.5	241^1^± 30.3	237.6^1^± 25.2	148.5 ± 22.3
Stiffness, N/mm	60.7^1^ ± 13.6	55.3^1^ ± 17.0	52.1^1^ ± 8.6	30.5 ± 7.2
Cyclic Loading displacement, mm				
100 cycles	0.7 ± 0.2	0.7 ± 0.2	0.7 ± 0.2	0.8 ± 0.2
300 cycles	1.1 ± 0.3	1.0 ± 0.2	1.1 ± 0.3	1.1 ± 0.3
500 cycles	1.3 ± 0.4	1.1 ± 0.2	1.3 ± 0.3	1.3 ± 0.3

Values are expressed as mean ± standard deviation

^1^Significantly different from Double Horizontal (*P* < 0.05)

^2^Significantly different from Hybrid (*P* < 0.05)

### Ultimate failure load

**Fig. 3:**
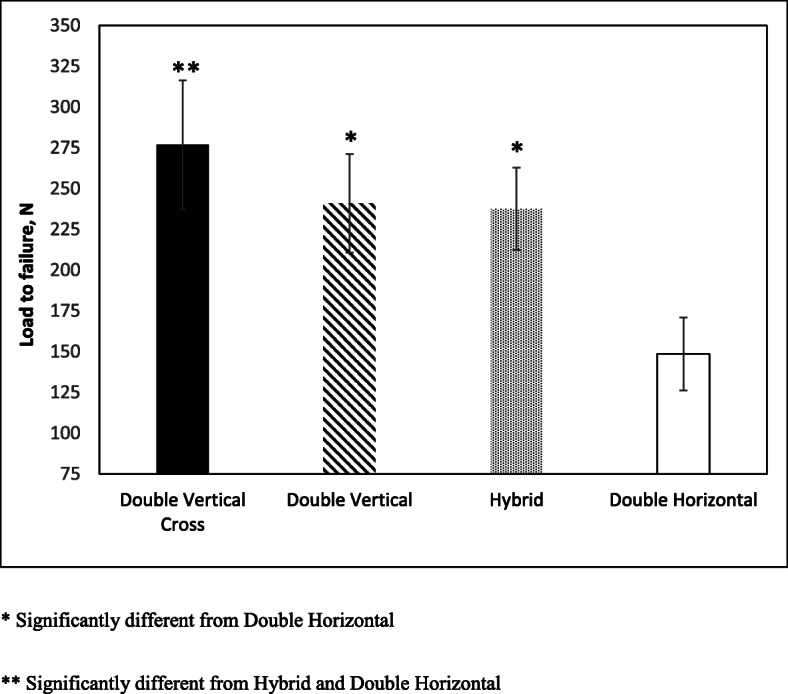
Load to failure depicted for all four repair groups. * Significantly different from Double Horizontal. ** Significantly different from Hybrid and Double Horizontal

All data (displacement, failure load, and stiffness) were distributed normally (*p* > .05 for all cases). Statistically significant difference was observed when comparing the failure load across all groups (*p* < .001). As shown in Fig. 3, the DV (241 ± 30.3 N, *p* < .001), the DVX (276.8 ± 39.5 N, *p* < .001) and Hybrid (237.6 ± 25.2 N, *p* < .001) groups had significantly higher average ultimate failure load compared to the DH group (148.5 ± 22.3 N). Furthermore, the average failure load of the DVX group is significantly higher than that of the Hybrid group by 39.3 N (*p* < .01). However, no significant differences in the average failure load were detected between the DV, DVX and Hybrid groups.

### Stiffness

**Fig. 4:**
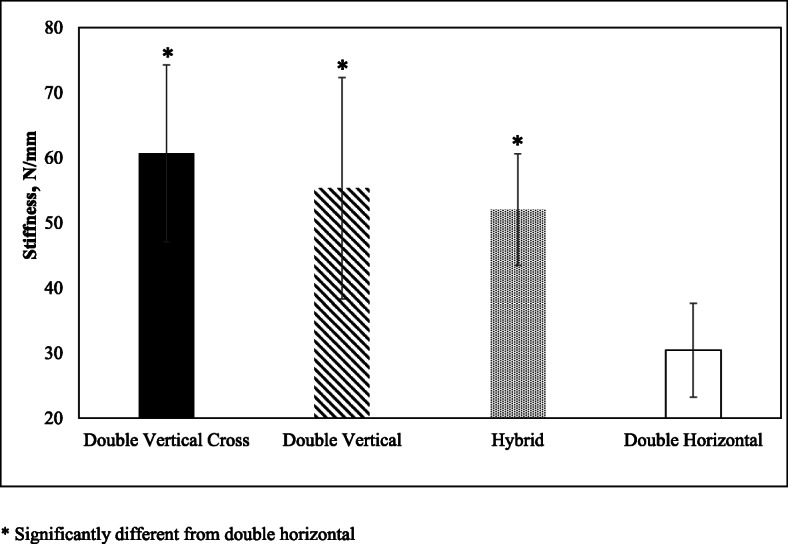
Stiffness depicted for all four repair groups. * Significantly different from double horizontal

Similarly, statistically significant difference in stiffness was observed across all groups (*p* < .001). Shown in Fig. 4, all the DVX (60.7 ± 13.6 N/mm, *p* < .001), Hybrid (52.1 ± 8.6 N/mm, *p* < .01) and the DV (55.3 ± 17.0 N/mm, *p* < .01) groups had significantly higher average stiffness than the DH group (30.5 ± 7.2 N/mm). However, even though the DVX group had higher average of stiffness than the Hybrid and DV groups, the differences were not significant.

### Displacement during cyclic loading

**Fig. 5:**
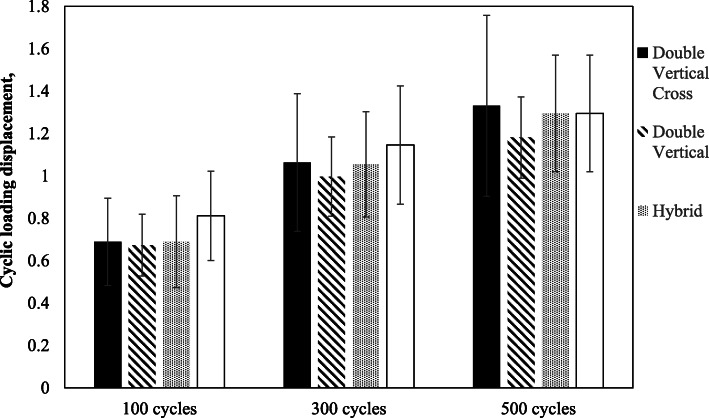
Displacement after 100, 300, and 500 cycles depicted across four repair groups

In Fig. 5, we observed a trend of larger displacement with increased cycles of loading, irrespective of the suturing techniques. At 100, 300 and 500 cycles, the average displacement after cyclic loading in the Hybrid group was always the largest among the four groups. In contrast, the average displacement in the DV group was always the smallest among the four groups. However, the group differences did not reach statistical significance at 100 cycles (*p* = .42), at 300 cycles (*p* = .68), and at 500 cycles (*p* = .70).

### Mode of failure

Tissue failure was observed across all specimens tested, and none of the failure resulted from suture or knot rupture.

## Discussion

In this study, we found that the three vertical suture techniques showed significantly higher failure load and stiffness when compared with double horizontal suture technique in the repair of complete radial tear of medial meniscus. Therefore, the results of our study support our hypotheses that all three meniscus repair techniques were biomechanically superior to the conventional double horizontal suture technique.

The conventional inside-out and outside-in approaches can only pass horizontal sutures that are parallel to the tibia plateau. As a result, previous studies also reported that the inside-out/outside-in double horizontal sutures were associated with additional surgical incision at the posterior capsule, long surgical time, frequent neurovascular complications, restricted range of motion and weight-bearing during post-operation rehabilitation [1, 15, 32, 43]. The advancement of all-inside devices allows more complex and stronger meniscus repair. For example, Beamer et al. [6] used a meniscus repair device to pass sutures vertically through the meniscus, which works similarly to the Knee Scorpion device used in our study. They found that single vertical suture repair showed better fixation than single horizontal loop repair carried out by inside-out technique [6]. Similar to the findings of Beamer et al’s study, the double vertical technique in our study employed two vertically oriented suture threads and yielded stronger fixation than the double horizontal technique. It also had a mean failure load and stiffness that were more than twice the values of the single vertical sutures in Beamer et al’s study [6]. Furthermore, compared to the double horizontal cross configuration in the study by Matsubara et al. (which yielded a ultimate failure load of 78.9 ± 19.3 N and stiffness of 8.0 ± 1.5 N/mm) and the cross tie-grip technique that deploys parallel sutures but modified configurations in the study by Nakanishi et al. (which yielded a ultimate failure load of 154.9 ± 29.0 N) [28, 30], our double vertical and double vertical cross configuration yielded much stronger repair and better fixation. We reason that the double vertical and double vertical cross techniques both employ vertical stitches that are perpendicular to the radial fibrils, thus binding them more tightly at the site of tear [6].

The Hybrid configuration composed of one vertical loop at the superior level and one horizontal loop at the inferior level, which had comparable strength to the Double Vertical and Double Vertical Cross, but stronger and stiffer than the Double horizontal technique. In the Hybrid configuration, two loops of sutures form a 90-degree cross inside the meniscus and provide three dimensional primary stabilities against rotatory forces in more complex physiological conditions. However, this was not able to be confirmed in our study, due to the fact that the mechanical testing was limited to the axial force loaded on a single plane.

The primary stability of the repaired meniscus is the most important goal to achieve in successful meniscal repair [28]. To achieve this goal, Herbort et al. [20] found that double-loop horizontal sutures in radial meniscus lesion repair showed greater failure load, less gap formation, and provided stiffer construct than single-loop horizontal suture repair. Matsubara et al. [28] showed that the two horizontal sutures loops forming a cross were biomechanically superior to double horizontal sutures technique in radial meniscus lesion repair. They proved that sutures oblique to circumferential collagen fibrils showed better fixation than those parallel to circumferential fibrils. Current literature emphasizes the importance of suture orientation, number of suture loops, fixation location in radial meniscus lesion repair, given available instruments. The two repair techniques that we proposed in this study integrated the elements contributing to a stronger repair construct and the results of biomechanical testing supported that vertical sutures offered better primary stability supported by the microarchitecture of meniscus. In general, we found that adding vertical sutures significantly improved the strength of the repair construct, because, as speculated by Beamer et al., vertical suture loop perpendicular to the radial tear effectively encircles the radial collagen fibrils [6]. Thus, sutures oblique to the circumferential fibrils are able to resist higher forces.

Furthermore, in the hybrid technique, the horizontal suture loop is practically easier to perform than vertical suture and it can be carried out by either inside-out or outside-in approach. Especially in the anterior or posterior corner of the joint, it is difficult for the vertical suture device to repair the tear.

The gap formation in current study did not differ significantly across all four groups, which was not found in previous studies with similar settings [6, 28, 33]. It may be because the knots were tied very tightly using a knot pusher in our study. The loading force and number of cycles of the cyclic adopted from previous studies did not produce gaps large enough to detect differences across different groups.

Unlike previous studies that various failure modes were reported [6, 20, 24, 33], tissue failure was uniformly noted in our study, which showed that the suture material selected was strong and knots were secure. Furthermore, in this study we only used medial menisci for a better control of the confounding factors. Compared to lateral meniscal tear, the medial meniscus has a higher risk of injuries, because the medial meniscus is attached more tightly to the joint capsule and surrounding tissues, especially the posterior horn is even less mobile, thus more susceptible to injury [31, 44].

Our study has several limitations. Although porcine menisci are similar in shape and function with human, the tissue is not a perfect surrogate. Porcine menisci are thicker, denser and smaller than human menisci and thus may not reflect accurate biomechanical properties [6, 21]. The porcine menisci were harvested from same-aged pigs allowing testing its biomechanical behaviors in a standardized fashion. Thus, we can exclude the confounding factors of highly variable degenerative menisci from the cadaver donors. Previous studies used porcine menisci and found it a good biomechanical model [20, 34, 36, 38]. The study aimed to study the biomechanical properties of repair techniques for radial meniscal lesion. To eliminate confounding factors, a complete radial tear was made and axial force perpendicular to the tear was applied. However, this setting did not reflect the physiological conditions, in which compression, tension, and shear forces apply to the meniscus simultaneously. Furthermore, the repair knots were tied by hands in an open fashion uniformly for all specimens, consequently very small gaps formed after the completion of cyclic loading. This study design simulates the immediate post-operation rehabilitation, where there is no healing and the repair is more vulnerable to damage. Despite that all three techniques showed significantly higher strength and stiffness, it is still unknown that to what degree strength and stiffness of the repaired construct will yield ideal clinical outcomes.

## Conclusion

The two techniques for repairing radial meniscus tear developed and proposed – the double vertical cross and the hybrid suture techniques, as well as the double vertical suture, are superior than the conventional double horizontal repair technique in terms of strength and stiffness.

## Data Availability

The datasets used and/or analyzed during the current study are available from the corresponding author on reasonable request.

## Abbreviations

DH: Double horizontal
DV: Double vertical
DVX: Double vertical cross
